# Liberate Abstract Garbage Collection from the Stack by Decomposing the Heap

**DOI:** 10.1007/978-3-030-44914-8_8

**Published:** 2020-04-18

**Authors:** Kimball Germane, Michael D. Adams

**Affiliations:** ETH Zurich, Zurich, Switzerland; 9 Brigham Young University, Provo, UT USA; 10 University of Michigan, Ann Arbor, MI USA

**Keywords:** Control-Flow Analysis, Abstract Garbage Collection, Pushdown Systems

## Abstract

Abstract garbage collection and the use of pushdown systems each enhance the precision of control-flow analysis (CFA). However, their respective needs conflict: abstract garbage collection requires the stack but pushdown systems obscure it. Though several existing techniques address this conflict, none take full advantage of the underlying interplay. In this paper, we dissolve this conflict with a technique which exploits the precision of pushdown systems to decompose the heap across the continuation.This technique liberates abstract garbage collection from the stack, increasing its effectiveness and the compositionality of its host analysis. We generalize our approach to apply compositional treatment to abstract timestamps which induces the context abstraction of *m*-CFA, an abstraction more precise than *k*-CFA’s for many common programming patterns.

### 1 Introduction

Among the many enhancements available to improve the precision of control-flow analysis (CFA), abstract garbage collection and pushdown models of control flow stand out as particularly effective ones. But their combination is non-trivial.

Abstract garbage collection (GC) [10] is the result of applying standard GC—which calculates the heap data reachable from a root set derived from a given environment and continuation—to an abstract semantics. Though it operates in the same way as *concrete* GC, abstract GC has a different effect on the semantics to which it's applied. Concrete GC is semantically irrelevant in that it has no effect on a program's observable behavior.[3] Abstract GC, on the other hand, is semantically relevant in that, by eliminating some merging in the abstract heap, it prevents a utilizing CFA from conflating some distinct heap data. In the setting of a higher-order language, where data can represent control, this superior approximation of data translates to a superior approximation of control as well, manifest by the CFA exploring fewer infeasible execution paths.

Pushdown models of control flow [16, 3] encode the call–return relation of a program's flow of execution as precisely as an unbounded control stack would

[3] It is irrelevant only if space consumption is unobservable, as is typical.

allow. Consequently, and in contrast to the finite-state models which preceded them, pushdown models enable a utilizing CFA—a *stack-precise* CFA—to avoid relating a given return to any but its originating call. Thus, pushdown models also induce CFAs which explore fewer infeasible execution paths.

Not only do abstract GC and pushdown systems each enhance the control precision of CFA, they also appear to do so in complementary ways. Is it possible for a CFA to use both and gain the benefits of each? This question's answer is not immediate, as these techniques have competing requirements: abstract GC must examine the stack to extract the root set of reachability but the use of pushdown models obscures the control stack to the abstract semantics.

This question has been addressed by two techniques: The first *introspective* technique [4] introduces a primitive operation into the analyzing machine which introspects the stack and delivers the set of frames which may be live; this technique has a variety of alternative formulations, some of which alter its complexity–precision profile [8, 7]. The second technique [1], which modifies the first to work with definitional interpreters, dictates that the analyzer implement a set-passing style abstract semantics where each passed set contains the heap addresses present in the continuation at that point. Each of these techniques reconciles the competing requirements of abstract GC and pushdown models of control flow and allows the utilizing CFA to enjoy the precision-enhancing benefits of both at once.

However, each of these techniques—hereafter referred to collectively as *pushdown GC*—yields a setting in which abstract GC and pushdown models of control flow merely coexist. In contrast, this paper prescribes a technique which *exploits* the pushdown model of control flow to enable a new mode of garbage collection—*compositional garbage collection*—which does not require the ability to inspect the continuation.

The key observation is that, in a stack-precise CFA, the heap present at the point of a call is in scope at the point of its return. Thus, the analysis can offload some of the contents of the callee's heap to the caller's—in particular, the data irrelevant to the callee's execution. When this offloading is performed, the final heap of the callee (just as it returns) is incomplete with respect to subsequent execution. But, since the caller's heap is in scope at this point, the analysis can reconstitute the subsequent heap by combining the caller's heap with the callee's final heap.

The data *relevant* to the callee's execution is the data reachable from its local environment and excludes the data reachable from its continuation alone. Offloading heap data, then, consists of GC-ing each callee's heap with respect to its local environment only. When one applies this practice consistently to all calls, one associates with each active call not a heap but a *heap fragment*, effectively decomposing the heap across the continuation. As we will show, careful separation and combination of these heap fragments can perfectly simulate the presence of the full heap.

This liberation of GC from the continuation has several consequences for the host CFA.

1. It simplifies both the formalization and implementation of the host CFA, since it can omit the relatively complex machinery to ensure the continuation-resident addresses are at hand.
2. It reduces the host CFA's workload by not requiring it to traverse full heaps. Earl *et al.* [4] observe that traversal of large heaps observably increases analysis time.
3. It recovers *context irrelevance* in the host CFA's semantics, a property we discuss more in Section 3.4 and Section 6.1.
4. It enables purely-local execution summaries which makes memoization much more effective.

In sum, relative to pushdown GC, compositional GC offers quantitative benefits to the host CFA, being strictly more powerful, as well as qualitative.

#### 1.1 Examples

Let's look at an example where compositional GC makes memoization more effective. Consider the following Scheme program

```
(let* ([id (lambda (x) x)]
       [y (id 42)]
       [z (id y)])
  (+ y z))
```

which calls `id` twice, each time on 42.

We would hope that a CFA would be able to memoize its analysis of the first call and, upon recognizing that the second call is semantically-identical, re-use its results. However, contemporary CFAs will not because each call is made with a different heap—the second call's heap includes a binding for `y` that the first's doesn't. Moreover, this distinction persists even with pushdown GC since `y`'s binding is needed to continue execution after the call. Since CFAs have no means but reachability to determine what is relevant to a given execution point, and since what is relevant constitutes a memoization key, pushdown GC is too weak to identify these two calls.

In contrast, a CFA with compositional GC produces a heap fragment for each call which is closed over only data reachable from the local environment—for a call, the procedure and argument values themselves. Accordingly, from its perspective, these two calls are identical and specify a single memoization key.

Now let's look at an example where compositional GC keeps co-live bindings of the same variable distinct. Consider the following Scheme program

```
(letrec ([f (lambda (x)
              (if (prime? x)
                (let ([y (f (+ x 1))])
                  (+ x y))
                x))])
  (f 2))
```

which defines and calls a recursive procedure `f`.

Concrete evaluation of this program proceeds first calls `f` with 2, and then 3, and then 4, returning 4, and then $3 + 4 = 7$ and then $2 + 7 = 9$. The procedure `f` is properly recursive—so these calls are nested—and, after `f` is called with 4 but before it returns, three distinct bindings of `x` are live. Moreover, since each binding of `x` is needed until its binding call returns, each is continuation-reachable and therefore not claimed by GC. These facts and limitations translate to the analysis setting: a CFA will discover multiple co-live bindings of `x` which persist in the face of pushdown GC. Consequently, even with pushdown GC, a CFA will in general join these bindings to some degree, concluding that `x` can be 2 whenever it can be 3 and can be 3 whenever it can be 4.

In constrast, just before a CFA with compositional GC performs each call to `f`, it GCs with respect to the operator and argument values which, in each case, consist of the closure of `f` (which reaches only itself in the heap) and a number (which doesn't reach anything). Thus, each binding to `x` is the first in its respective heap fragment and doesn't interfere with the live bindings of `x` in other heap fragments. Using a numeric abstraction in which arithmetic operations propagate but do not introduce approximation [1], a CFA with compositional GC will produce an exact answer (whereas one with pushdown GC will not).

#### 1.2 Generalizing the Approach

The conventional treatment of the heap by CFA is to thread it through execution, allowing it to evolve as it goes. In contrast, compositional GC advocates that the CFA treat the heap with the same discipline that it treats the environment: saved at the evaluation of a subexpression and restored when its evaluation completes and its value is delivered. That is, compositional GC is achieved by, in effect, treating the heap compositionally.

What happens if we impose the same compositional discipline on other threaded components, such as the timestamp? In that case, we move from the last-$k$-call-sites[4] context abstraction of $k$-CFA [14] to the top-$m$-stack-frames[5] context abstraction of $m$-CFA [11] This appearance of $m$-CFA's abstraction in a stack-precise CFA is the first such, to our knowledge.

With compositional treatment of both the heap and timestamp, we arrive at a stack-precise CFA which treats each of its components compositionally. This treatment also leads to a CFA closer to being compositional in the sense that the analysis of a compound expression is a function of the analyses of its constituent parts. Accordingly, we refer to such a stack-precise CFA as a *compositional control-flow analysis.*

The remainder of the paper is as follows. We first introduce the syntax of the language we will use throughout the paper in Section 2. We then discuss the enhancements of perfect stack precision, garbage collection, and their combination in Section 3. We then proceed through a series of semantics which transition

[4] as in, *most-recent* $k$ call sites
[5] as in, *youngest* $m$ stack frames from a threaded heap to a compositional, garbage-collected heap in Section 4. We then abstract the compositional semantics to obtain our CFA in Section 5. We discuss the ramifications of the compositional treatment of each of the heap and abstract time in Section 6. We finally discuss related work in Section 7 and conclusions and future work in Section 8.

**Note** In the remainder of the paper, we use the standard term *store* to refer to the analysis component which models the heap. Thus, we will describe our technique as, e.g., *treating stores compositionally.*

### 2 A-Normal Form λ-Calculus

For presentation, we keep the language small: we use a unary λ-calculus in $\mathcal{A}$-normal form [5], the grammar of which is given below.

$$\begin{aligned} \mathit{Exp} \ni e &::= \mathit{ce} \mid \mathsf{let}\, x = \mathit{ce} \,\mathsf{in}\, e \\ \mathit{CExp} \ni \mathit{ce} &::= \mathit{ae} \mid (\mathit{ae}_0\ \mathit{ae}_1) \mid \mathsf{set!}\, x\ \mathit{ae} \\ \mathit{AExp} \ni \mathit{ae} &::= x \mid \lambda x.e \\ \mathit{Var} \ni x & \quad \text{[an infinite set of variables]} \end{aligned}$$

A proper expression $e$ is a *call expression* $ce$ or a let-expression, which binds a variable to the result of a call expression. (Restricting the bound expression to a call expression prevents let-expressions from nesting there, a hallmark of $\mathcal{A}$-normal form.) A call expression $ce$ is an *atomic expression* $ae$, an application, or a set!-expression. An atomic expression $ae$ is a variable reference or a $\lambda$ abstraction.

Atomic expressions are trivial [13]. We include set!-expressions to produce mutative effects that must be threaded through evaluation. (The approach we present in this paper can also handle more-general forms of mutation, such as boxes.) For our purposes, we consider a set!-expression "serious" [13] since it has an effect on the store.

A program is a closed expression; we assume (without loss of generality) that programs are alphatised—that is, that each bound variable has a distinct name.

Expressions of the form $(ae_0\ ae_1)$ for some $ae_0$ and $ae_1$ constitute the set *App*; similarly, expressions of the form $\lambda x.e$ for some $x$ and $e$ constitute the set *Lam*.

### 3 Background

In this section, we review abstract garbage collection and the $k$-CFA context abstraction. We begin by introducing a small-step concrete semantics which defines the ground truth of evaluation.

#### 3.1 Semantic Domains

First, we introduce some semantic components that we will use heavily throughout the rest of the paper.

$$v \in \mathit{Val} = \mathit{Lam} \times \mathit{Env} \qquad \rho \in \mathit{Env} = \mathit{Var} \rightharpoonup \mathit{Time}$$
$$t \in \mathit{Time} = \mathit{App}^* \qquad a \in \mathit{Address} = \mathit{Var} \times \mathit{Time}$$
$$\sigma \in \mathit{Store} = \mathit{Address} \rightharpoonup \mathit{Val} \qquad \kappa \in \mathit{Cont} ::= \mathsf{mt} \mid \mathsf{lt}(x, \rho, e, \kappa)$$

A value $v$ is closure, a pair of a $\lambda$ abstraction and an environment which closes it. An environment $\rho$ is a finite map from each variable $x$ to a time $t$; a time $t$ is a finite sequence of call sites. Let $\rho|_e$ denote the restriction of the domain of the environment $\rho$ to the free variables of $e$. An address $a$ is a pair of a variable and time and a store $\sigma$ is a map from addresses to values. A continuation $\kappa$ is either the empty continuation or the continuation of a $\mathsf{let}$ binding.

#### 3.2 Concrete Semantics

We define our concrete semantics as a small-step relation over abstract machine states. The state space of our machine is given formally as follows.

$$\begin{aligned} \varsigma \in \mathit{State} &= \mathit{Eval} + \mathit{Apply} \\ \varsigma_{\mathsf{ev}} \in \mathit{Eval} &= \mathit{Exp} \times \mathit{Env} \times \mathit{Store} \times \mathit{Cont} \times \mathit{Time} \\ \varsigma_{\mathsf{ap}} \in \mathit{Apply} &= \mathit{Val} \times \mathit{Store} \times \mathit{Cont} \times \mathit{Time} \end{aligned}$$

Machine states come in two variants. An *Eval* machine state represents a point in execution in which an expression will be evaluated; it contains registers for an expression $e$, its closing environment $\rho$, the store $\sigma$ (modelling the heap), the continuation $\kappa$ (modelling the stack), and the time $t$. An *Apply* machine state represents a point in execution at which a value is in hand and must be delivered to the continuation; it contains registers for the value $v$ to deliver, the store $\sigma$, the continuation $\kappa$, and the time $t$.

Figure 1 contains the definitions of two relations over machine states, the union of which constitutes the small-step relation. The $\rightarrow_{\mathsf{ev}}$ relation transitions an *Eval* state to its successor. The Let rule pushes a continuation frame to save the bound variable, environment, and body expression. The resultant *Eval* state is poised to evaluate the bound expression $ce$. The Call rule first uses $\mathsf{aeval}$ defined

$$\mathsf{aeval}(\sigma, \rho, x) = \sigma(x, \rho(x)) \qquad \mathsf{aeval}(\sigma, \rho, \lambda x.e) = (\lambda x.e, \rho|_{\lambda x.e})$$

to obtain values for each of the operator and argument. It then increments the time, extends the store and environment with the incremented time, and arranges evaluation of the operator body at the incremented time. The Set! rule remaps a location in the store designated by a given variable (which is resolved in the environment) to a value obtained by $\mathsf{aeval}$. It returns the identity function.

$$\frac{\textsc{Let}}{\mathsf{ev}(\mathsf{let}\, x = ce \,\mathsf{in}\, e, \rho, \sigma, \kappa, t) \rightarrow_{\mathsf{ev}} \mathsf{ev}(ce, \rho, \sigma, \mathsf{lt}(x, \rho, e, \kappa), t)}$$

$$\textsc{Call}\quad \frac{(\lambda x.e, \rho') = \mathsf{aeval}(\sigma, \rho, ae_0) \qquad v = \mathsf{aeval}(\sigma, \rho, ae_1) \qquad t' = (ae_0\ ae_1) :: t \qquad \sigma' = \sigma[(x, t') \mapsto v] \qquad \rho'' = \rho'[x \mapsto t']}{\mathsf{ev}((ae_0\ ae_1), \rho, \sigma, \kappa, t) \rightarrow_{\mathsf{ev}} \mathsf{ev}(e, \rho'', \sigma', \kappa, t')}$$

$$\textsc{Set!}\quad \frac{v = \mathsf{aeval}(\sigma, \rho, ae) \qquad a = (x, \rho(x)) \qquad \sigma' = \sigma[a \mapsto v]}{\mathsf{ev}(\mathsf{set!}\, x\ ae, \rho, \sigma, \kappa, t) \rightarrow_{\mathsf{ev}} \mathsf{ap}((\lambda \mathsf{x}.\mathsf{x}, \bot), \sigma', \kappa, t)}$$

$$\textsc{Atomic}\quad \frac{v = \mathsf{aeval}(\sigma, \rho, ae)}{\mathsf{ev}(ae, \rho, \sigma, \kappa, t) \rightarrow_{\mathsf{ev}} \mathsf{ap}(v, \sigma, \kappa, t)} \qquad \textsc{Apply}\quad \frac{\rho' = \rho[x \mapsto t] \qquad \sigma' = \sigma[(x, t) \mapsto v]}{\mathsf{ap}(v, \sigma, \mathsf{lt}(x, \rho, e, \kappa), t) \rightarrow_{\mathsf{ap}} \mathsf{ev}(e, \rho', \sigma', \kappa, t)}$$

**Fig. 1.** Small-step abstract machine semantics

The Atomic rule evaluates an atomic expression. The Apply rule applies a continuation to a value, extending the environment and store and arranging for the evaluation of the let body.

We *inject* a program $pr$ into the initial evaluation state $\mathsf{ev}(pr, \bot, \bot, \mathsf{mt}, \langle\rangle)$ which arranges evaluation in the empty environment, empty store, halt continuation, and empty time.

**Adding Garbage Collection** At this point, we have a small-step relation defining execution by abstract machine and are perfectly positioned to apply, e.g., the Abstracting Abstract Machines (AAM) [15] recipe to *abstract* the semantics and thereby obtain a sound, computable CFA. Before doing so, however, we will extend our semantics to garbage-collect the store on each transition. This extension has no semantic effect in the concrete semantics but, as we will discuss, greatly increases the precision of the abstracted (or, simply, *abstract*) semantics.

We extend the semantics by defining two garbage collection transitions, one which collects an *Eval* state and one which collects an *Apply* state. Because our abstract machine explicitly models local environments, heaps (via stores), and stacks (via continuations), we can apply a copying collector to perform garbage collection.

First, we define a family root of metafunctions to extract the reachability root set from values, environments, and continuations.

$$\mathsf{root}_v(\lambda x.e, \rho) = \mathsf{root}_\rho(\rho) \qquad \mathsf{root}_\kappa(\mathsf{mt}) = \emptyset$$

$$\mathsf{root}_\rho(\rho) = \rho \qquad \mathsf{root}_\kappa(\mathsf{lt}(x, \rho, e, \kappa)) = \mathsf{root}_\rho(\rho|_e) \cup \mathsf{root}_\kappa(\kappa)$$

The $\mathsf{root}_v$ metafunction extracts the root addresses from a closure by using $\mathsf{root}_\rho$ to extract the root addresses from its environment. By the $\mathsf{root}_\rho$ metafunction, the root addresses of an environment are simply the variable–time pairs that define it—that is, the definition of $\mathsf{root}_\rho$ views its argument $\rho$ extensionally as a set of addresses. The $\mathsf{root}_\kappa$ metafunction extracts the root addresses from a continuation. The empty continuation has no root addresses whereas the root addresses of a non-empty continuation are those of its stored environment (restricted to the free variables of the expression it closes) combined with those of the continuation it extends.

Next, we define a reachability relation $\to_\sigma$ parameterized by a store $\sigma$ and over addresses by

$$a_0 \to_\sigma a_1 \Leftrightarrow a_1 \in \mathsf{root}_v(\sigma(a_0))$$

We then define the reachability of a root set with respect to a store

$$\mathcal{R}(\sigma, A) = \{a' : a \in A, a \to_\sigma^* a'\}$$

where $\to_\sigma^*$ is the reflexive, transitive closure of $\to_\sigma$. From here, we obtain the transitions

$$\textsc{GC-Eval}\quad \frac{A = \mathsf{root}_\rho(\rho|_e) \cup \mathsf{root}_\kappa(\kappa) \qquad \sigma' = \sigma|_{\mathcal{R}(\sigma,A)}}{\mathsf{ev}(e, \rho, \sigma, \kappa, t) \to_{\mathsf{GC}} \mathsf{ev}(e, \rho, \sigma', \kappa, t)}$$

$$\textsc{GC-Apply}\quad \frac{A = \mathsf{root}_v(v) \cup \mathsf{root}_\kappa(\kappa) \qquad \sigma' = \sigma|_{\mathcal{R}(\sigma,A)}}{\mathsf{ap}(v, \sigma, \kappa, t) \to_{\mathsf{GC}} \mathsf{ap}(v, \sigma', \kappa, t)}$$

where $\sigma|_{\mathcal{R}(\sigma,A)}$ is $\sigma$ restricted to the reachable addresses $\mathcal{R}(\sigma, A)$. We compose this garbage-collecting transition with each of $\to_{\mathsf{ev}}$ and $\to_{\mathsf{ap}}$. Altogether, the garbage-collecting semantics are given by $\to_{\mathrm{GC}} \circ[\to_{\mathsf{ev}} \cup \to_{\mathsf{ap}}]$.

#### 3.3 Abstracting Abstract Machines with Garbage Collection

Now that we have a small-step abstract machine semantics with GC, we are ready to apply the AAM recipe to obtain a sound, computable CFA with GC.

We apply the AAM recipe in two steps.

First, we refactor the state space so that all inductively-defined components are redirected through the store. Practically, this refactoring has the effect of allocating continuations in the store. For our semantics, this refactoring yields the state space $State_{SA}$ defined

$$\begin{aligned} State_{SA} &= Eval_{SA} + Apply_{SA} \\ Eval_{SA} &= Exp \times Env \times Store_{SA} \times ContAddr \times Time \\ Apply_{SA} &= Store_{SA} \times ContAddr \times Val \times Time \end{aligned}$$

in which a continuation address $\alpha \in ContAddr$ replaces the continuation drawn from *Cont*. The space of continuations becomes defined by

$$\kappa_{SA} \in Cont_{SA} ::= \mathsf{mt} \mid \mathsf{lt}(x, \rho, e, \alpha)$$

and of stores by

$$Store_{SA} = Address + ContAddr \rightharpoonup Val + Cont_{SA}$$

Not reflected in this structure is the typical constraint that an address $a$ will only ever locate a value and a continuation address $\alpha$ will only ever locate a continuation.

Second, we finitely partition the unbounded address space of the store and treat the constituent sets as abstract addresses (via some finite representative). Practically, this partitioning is achieved by limiting the time $t$ to at most $k$ call sites where $k$ becomes a parameter of the CFA (leading to the designation $k$-*CFA*). Any addresses which agree on the $k$-length prefix of their time component are identified and the finite representative for this set of addresses uses simply that prefix. Accordingly, we define an abstract time domain $\widehat{Time} = Time^{\leq k}$ and let it reverberate through the state space definitions, obtaining

$$\begin{aligned}
\widehat{State} &= \widehat{Eval} + \widehat{Apply} \\
\widehat{Eval} &= Exp \times \widehat{Env} \times \widehat{Store} \times \widehat{ContAddr} \times \widehat{Time} \\
\widehat{Apply} &= \widehat{Store} \times \widehat{ContAddr} \times \widehat{Val} \times \widehat{Time}
\end{aligned}$$

(in which we allow the definition of *ContAddr* to depend, directly or not, on that of *Time*).

Finitization of the address space is key to producing a computable CFA. Practically, however, it means that some values located previously by distinct addresses will after be located by the same abstract address. When this conflation occurs, the CFA must behave as if either access was intended; this behavior is manifested by non-deterministically choosing the value located by a particular address. Because our language is higher-order, this non-determinism also affects the control flows the CFA considers. This effect is evident in the Call rule defined

$$\textsc{Call}\quad \frac{(\lambda x.e, \hat{\rho}') \in \widehat{\mathsf{aeval}}(\hat{\sigma}, \hat{\rho}, ae_0) \qquad \hat{v} = \widehat{\mathsf{aeval}}(\hat{\sigma}, \hat{\rho}, ae_1) \qquad \hat{t}' = \lfloor (ae_0\ ae_1) :: \hat{t} \rfloor_k \qquad \hat{\sigma}' = \hat{\sigma}[(x, \hat{t}') \mapsto \hat{v}] \qquad \hat{\rho}'' = \hat{\rho}'[x \mapsto \hat{t}']}{\mathsf{ev}((ae_0\ ae_1), \hat{\rho}, \hat{\sigma}, \hat{\alpha}, \hat{t}) \to_{\mathsf{ev}} \mathsf{ev}(e, \hat{\rho}'', \hat{\sigma}', \hat{\alpha}, \hat{t}')}$$

which is structurally identical to that of the concrete semantics except in two respects:

1. The abstract evaluation of the operator $ae_0$ may yield multiple closures and the CFA considers the application of each. Due to the approximation finitization introduces, not every abstractly-applied closure will necessarily appear in a compatible call under the concrete semantics. Such closures, initiating spurious control paths, waste analysis effort and this waste compounds as the exploration of spurious paths leads to the discovery of yet more.
2. The abstract time component is limited to length at most $k$ (obtained by $\lfloor \cdot \rfloor_k$).

In short, a finite address space introduces a value approximation and, in a higher-order language such as ours, a control approximation as well.

While the strategy to store-allocate continuations facilitates the systematic abstraction process of AAM, it also imposes a similar approximation on the continuation space as it does the value space. In consequence, a CFA obtained by AAM approximates not only the value and control flow of the program, but the return flow as well. Return-flow approximation is manifest as a single abstract call returning to caller contexts that did not make that call.[6]

On the other hand, because the AAM abstraction process preserves the overall structure of the state space—in particular, the explicit models of the local environment, heap, and stack—applying GC to an abstract state is straightforward. In addition, GC in the abstract semantics improves precision and reduces the workload of the analyzer [10].

To see how GC improves precision, consider a 0CFA (that is, $[k = 1]$CFA) without GC of the Scheme program

```
(let* ([id (lambda (x) x)]
       [y (id 42)]
       [z (id 35)])
  z)
```

at the call `(id 42)`. As the abstract call is made, the abstract value 42 is stored an address $a$ derived from `x`. Once the call returns, the abstract value 42 still resides in the heap at $a$ which is now unreachable. However, as the abstract call `(id 35)` is made, the address $a$ is derived again (a consequence of the finite address space), and the abstract value 35 is merged with the abstract value 42 which persists at $a$. Since the value at $a$ is returned and becomes the result of the program, the CFA reports that the program results in either 42 or 35.

Now consider a 0CFA with GC of the same program. Once the call `(id 42)` returns and $a$ becomes unreachable, its heap entry is reaped by GC. The abstract call `(id 35)` then allocates the abstract value 35 at $a$ which is, from the allocator's perspective, a fresh heap cell. Consequently, the CFA precisely reports that the program results in 35.

The above example also illustrates how GC reduces the workload of the analyzer. Though we didn't call it out, when using a naive continuation allocator without GC, the abstract call `(id 35)` not only correctly returns to the continuation binding `z` but also spuriously returns to the continuation binding `y`. In this example, this spurious control (return) flow does no more damage to the precision of 0CFA's approximation of the final program result, but does cause it to explore infeasible control flows which damage the precision of the 0CFA's approximation of intermediate values. GC prevents the spurious flows in this example from arising at all; however, in general, it does not prevent all spurious return flows.

[6] P4F [6] uses a particular continuation allocator which is able to avoid return-flow approximation. However, the P4F technique applies only when the store is globally-widened and, in such a setting, no data ever becomes unreachable which renders GC completely ineffective.

#### 3.4 Stack-Precise CFA with Garbage Collection

In contrast to an AAM-derived analysis, a stack-precise CFA does not approximate the return flow of the program. A stack-precise CFA achieves this feat by modelling control flow with a pushdown system which allows it to precisely match returns with their corresponding calls. However, to do so, it requires full control of the continuation which we abide by factoring it out of the state space, obtaining

$$\begin{aligned} \mathit{State}_{PD} &= \mathit{Eval}_{PD} + \mathit{Apply}_{PD} \\ \mathit{Eval}_{PD} &= \mathit{Exp} \times \mathit{Env} \times \mathit{Store} \times \mathit{Time} \\ \mathit{Apply}_{PD} &= \mathit{Val} \times \mathit{Store} \times \mathit{Time} \end{aligned}$$

before we abstract it to produce a CFA. (Some CFAs factor the store out of machine states to be managed globally, part of *widening the store*. In a sense, factoring out the continuation is part of *widening the continuation*.) Without a continuation component, an $\mathit{Eval}_{PD}$ state is an *evaluation configuration* and an $\mathit{Apply}_{PD}$ state is an *evaluation result*. Except for the presence of the time component, $\mathit{State}_{PD}$ exhibits precisely the configuration and result shapes one finds in many stack-precise CFAs [17, 8, 1, 18].

However, factoring the continuation out and ceding control of it to the analysis presents an obstacle to abstract GC, which needs to extract the root set of reachable addresses from it. Earl *et al.* [4] developed a technique whereby the analysis could introspect the continuation and extract the root set of reachable addresses from the continuation. Johnson and Van Horn [8] reformulated this *incomplete* technique for an operational setting and offered a *complete*—albeit theoretically more-expensive—technique capable of more precision. Johnson *et al.* [7] unified these techniques within an expanded framework. Darais *et al.* [1] then showed that the *Abstracting Definitional Interpreters*-approach—currently the state of the art—is compatible with the complete technique by including the set of stack root addresses as a component in the evaluation configuration.

**Context Irrelevance** These techniques indeed reconcile the conflicting needs of GC and stack-precise control yielding an analysis which enjoys the precision-enhancing benefits of each. However, the addition of garbage collection causes the resultant analysis to violate *context irrelevance* [8], the property that the evaluation of a configuration is independent of its continuation. In terms of the concrete semantics of Section 3.2, context irrelevance is the property that $\mathsf{ev}(e, \rho, \sigma, \kappa, t) \to^{+} \mathsf{ap}(\sigma', \kappa, v)$ if and only if $\mathsf{ev}(e, \rho, \sigma, \kappa', t) \to^{+} \mathsf{ap}(\sigma', \kappa', v)$ for any $\kappa$ and $\kappa'$.

The incomplete and complete techniques to achieve stack-precise abstract GC each violate context irrelevance. Under the incomplete technique, abstract GC prevents spurious paths from being explored and changes the store yielded by those that are explored. Thus, the abstract evaluation of a configuration becomes dependent on (the root set of reachable addresses embedded in) its continuation. The complete technique, achieved by introducing the set of root addresses as a component in the evaluation configuration, vacuously restores context irrelevance by distinguishing otherwise-identical configurations based on the continuation. That is, the states $\mathsf{ev}(e, \rho, \sigma, \kappa, t)$ and $\mathsf{ev}(e, \rho, \sigma, \kappa', t)$ with identical configurations but distinct continuations become the continuation-less evaluation configurations $\mathsf{ev}(e, \rho, \sigma, A, t)$ and $\mathsf{ev}(e, \rho, \sigma, A', t)$ with distinct root address sets $A$ and $A'$. This address set is a close approximation of the continuation and effectively makes the control context relevant to evaluation.

#### 3.5 The $k$-CFA Context Abstraction

In the concrete semantics, the time component $t$ serves two purposes. The first purpose is to provide the allocator with a source of freshness, so that when the allocator must furnish a heap cell for a variable bound previously in execution, it is able to furnish a distinct one. Were freshness the only constraint on $t$, the *Time* domain could simply consist of $\mathbb{N}$. In anticipation of its role in the downstream CFA, the time component assumes a second purpose which is to capture some notion of the context in which execution is occurring. The hope is that the notion of context it captures is semantically meaningful so that, when an unbounded set of times are identified by the process of abstraction, each address, which is qualified by such an abstracted time, locates a semantically-coherent set of values.

To get a better idea of what notion of context our treatment of time captures, let's examine how our concrete semantics treats time, as dictated by $k$-CFA. Time begins as the empty sequence $\langle\rangle$. It is passed unchanged across all *Eval* transitions, save one, and the *Apply* transition. The exception is the Call transition, which instead passes the (at-most-)$k$-length prefix of the application prepended to the incoming time. Hence, the $k$-CFA context abstraction is the $k$-most-recent calls made in execution history.

In Section 6.2, we consider the ramifications of threading the time component through evaluation and compare it to an alternative treatment.

### 4 From Threaded to Compositional Stores

In this section, we present a series of four semantics that gradually transition from a threaded treatment of stores without GC to a compositional treatment of stores with GC. We define each of these semantics in terms of big-step judgments of (or close to) the form $\sigma, \rho, t \vdash e \Downarrow (v, \sigma')$. This judgment expresses that the *evaluation configuration* consisting of the expression $e$ under the store $\sigma$, environment $\rho$, and timestamp $t$ evaluates to the *evaluation result* consisting of the value $v$ and the store $\sigma'$. When discussing the evaluation of $e$, we will refer to $\sigma$ as the incoming store and $\sigma'$ as the resultant store. We will also refer to the time component $t$ as the *binding context* since, in the big-step semantics, its connection to the history of execution becomes more distant.

Formulating our semantics in big-step style offers two advantages to our setting: First, we can readily express them by big-step definitional interpreters at which point we can apply systematic abstraction techniques [1, 18] to obtain corresponding CFAs exhibiting perfect stack precision. Second, they emphasize the availability of the configuration store at the delivery point of the evaluation result; this availability is crucial to our ability to shift to a compositional treatment of the store.

### 4.1 Threaded-Store Semantics

To orient ourselves to the big-step setting, we present the reference semantics for our language in big-step style in Figure 2. This reference semantics is equivalent to the reference semantics given in small-step style in Section 3.2 except that there is no corresponding Apply rule; its responsibility—to deliver a value to a continuation—is handled implicitly by the big-step formulation. In terms of big-step semantics, this reference semantics is characterized by the threading of the store through each rule; the resultant store of evaluation is the configuration store plus the allocation and mutation incurred during evaluation. Hence, we refer to this semantics as the *threaded-store* semantics. We use natural numbers as store subscripts in each rule to emphasize the store's monotonic increase.

Let

$$\frac{\begin{array}{c}\sigma_0, \rho, t \vdash ce \Downarrow (v_0, \sigma_1) \\ \rho' = \rho[x \mapsto t] \qquad \sigma_2 = \sigma_1[(x, t) \mapsto v_0] \qquad \sigma_2, \rho', t \vdash e \Downarrow (v, \sigma_3)\end{array}}{\sigma_0, \rho, t \vdash \mathsf{let}\, x = ce \,\mathsf{in}\, e \Downarrow (v, \sigma_3)}$$

Call

$$\frac{\begin{array}{c}((\lambda x.e, \rho_0), \sigma_1) = \mathsf{aeval}(\sigma_0, \rho, ae_0) \\ (v_1, \sigma_2) = \mathsf{aeval}(\sigma_1, \rho, ae_1) \qquad t' = (ae_0\ ae_1) :: t \\ \rho_1 = \rho_0[x \mapsto t'] \qquad \sigma_3 = \sigma_2[(x, t') \mapsto v_1] \qquad \sigma_3, \rho_1, t' \vdash e \Downarrow (v, \sigma_4)\end{array}}{\sigma_0, \rho, t \vdash (ae_0\ ae_1) \Downarrow (v, \sigma_4)}$$

Set!

$$\frac{(v, \sigma_1) = \mathsf{aeval}(\sigma_0, \rho, ae) \qquad \sigma_1 = \sigma_0[(x, \rho(x)) \mapsto v]}{\sigma_0, \rho, t \vdash \mathsf{set!}\, x\ ae \Downarrow ((\lambda \mathtt{x}.\mathtt{x}, \bot), \sigma_1)}$$

Atomic

$$\frac{}{\sigma, \rho, t \vdash ae \Downarrow \mathsf{aeval}(\sigma, \rho, ae)}$$

**Fig. 2.** The threaded-store semantics

A program *pr* is evaluated in an initial configuration with an empty store $\bot$, an empty environment $\bot$, and an empty binding context $\langle\rangle$. In such a configuration, *pr* evaluates to a value $v$ if $\bot, \bot, \langle\rangle \vdash pr \Downarrow (v, \sigma)$.

The Let rule evaluates the bound call expression *ce* under the incoming environment and store. If evaluation results in a value–store pair, this incoming environment is extended with a binding derived from the bound variable and incoming binding context.[7] The resultant store is extended with mapping from that binding to the resultant value. The body expression is evaluated under the extended environment and store and its result becomes that of the overall expression.

Contrasting the treatment of the environment and the store by the LET rule is instructive. On the one hand, the environment is treated compositionally: the incoming environment of evaluation is restored and extended after evaluation of the bound value. On the other hand, the store is treated non-compositionally: the store resulting from the evaluation of the bound expression is extended after it has accumulated the effects of its evaluation.

Under this criteria, we classify the treatment of the binding context as *compositional* rather than *threaded*. This compositional treatment departs from typical practice of CFA and is the first such treatment in a stack-precise CFA to our knowledge. In Section 6.2, we examine the ramifications of this treatment.

The CALL rule evaluates the atomic expressions $ae_0$ and $ae_1$ for the operator and argument, respectively. It then derives a new binding context, extends the environment and store with a binding using that context, and evaluates the operator body under the extended environment, store, and derived binding context. The result of evaluation the body is that of the overall expression.

The SET! rule evaluates the atomic body expression $ae$ and updates the binding of the referenced variable in the store. Its result is the identity function paired with the updated store.

The ATOMIC rule evaluates an atomic expression $ae$ using the aeval atomic evaluation metafunction. Foreshadowing the succeeding semantics, we define aeval to return a pair of its calculated value and the given store. In this semantics, the store is passed through unmodified; in forthcoming semantics, it will be altered according to the calculated value. Atomic evaluation is unchanged from the small-step semantics:

$$\mathsf{aeval}(\sigma, \rho, x) = (\sigma(x, \rho(x)), \sigma) \qquad \mathsf{aeval}(\sigma, \rho, \lambda x.e) = ((\lambda x.e, \rho|_{\lambda x.e}), \sigma)$$

### 4.2 Threaded-Store Semantics with Effect Log

The second semantics enhances the reference semantics with an *effect log* $\xi$ which explicitly records the allocation and mutation that occurs through evaluation. The effect log is considered part of the evaluation result; accordingly the *effect log semantics* are in terms of judgments of the form $\sigma, \rho, t \vdash e \Downarrow_! (v, \sigma'), \xi$. Figure 3 presents the effect log semantics, identical to the reference semantics except for (1) the addition of the effect log and (2) the use of the metavariable $a$ to denote an address $(x,t)$. (This usage persists in all subsequent semantics as well.)

The effect log is represented by a function from store to store. The definition of each log is given by either a literal identity function, a use of the $\mathsf{extend}_{log}$

[7] Because the program is alphatised, the binding of a let-bound variable in a particular calling context will not interfere with the binding of any other variable.

LET
$$\frac{\sigma_0, \rho, t \vdash ce \Downarrow_! (v_0, \sigma_1), \xi_0 \qquad \rho' = \rho[x \mapsto t] \qquad \sigma_2 = \sigma_1[(x,t) \mapsto v_0] \qquad \sigma_2, \rho', t \vdash e \Downarrow_! (v, \sigma_3), \xi_1}{\sigma_0, \rho, t \vdash \mathsf{let}\, x = ce\, \mathsf{in}\, e \Downarrow_! (v, \sigma_3), \xi_1 \circ \mathsf{extend}_{log}((x,t), v_0, \sigma_1) \circ \xi_0}$$

CALL
$$\frac{((\lambda x.e, \rho_0), \sigma_1) = \mathsf{aeval}(\sigma_0, \rho, ae_0) \qquad (v_1, \sigma_2) = \mathsf{aeval}(\sigma_1, \rho, ae_1) \qquad t' = (ae_0\; ae_1) :: t \qquad \rho_1 = \rho_0[x \mapsto t'] \qquad \sigma_3 = \sigma_2[(x,t') \mapsto v_1] \qquad \sigma_3, \rho_1, t' \vdash e \Downarrow_! (v, \sigma_4), \xi}{\sigma_0, \rho, t \vdash (ae_0\; ae_1) \Downarrow_! (v, \sigma_4), \xi \circ \mathsf{extend}_{log}((x,t'), v_1, \sigma_2)}$$

SET!
$$\frac{(v, \sigma_1) = \mathsf{aeval}(\sigma_0, \rho, ae) \qquad a = (x, \rho(x)) \qquad \sigma_1 = \sigma_0[a \mapsto v]}{\sigma_0, \rho, t \vdash \mathsf{set!}\, x\; ae \Downarrow_! ((\lambda \mathtt{x}.\mathtt{x}, \bot), \sigma_1), \mathsf{extend}_{log}(a, v, \sigma_1)}$$

ATOMIC
$$\frac{}{\sigma, \rho, t \vdash ae \Downarrow_! \mathsf{aeval}(\sigma, \rho, ae), \lambda\sigma.\sigma}$$

**Fig. 3.** Threaded-store semantics with an effect log metafunction, or the composition of effect logs. The $\mathsf{extend}_{log}$ metafunction is defined

$$\mathsf{extend}_{log}(a, v, \sigma') = \lambda\sigma.\sigma[a \mapsto v] \cup \sigma'$$

where the union of the extended store $\sigma[a \mapsto v]$ and the value-associated store $\sigma'$ treats each store extensionally as a set of pairs but the result is always a function—i.e. any given address is paired with at most one value. The effect log of the ATOMIC rule is the identity function, reflecting that no allocation or mutation is performed when evaluating an atomic expression. The effect log of the SET! rule is constructed by the metafunction $\mathsf{extend}_{log}$; the store argument to $\mathsf{extend}_{log}$ is the store *after* the mutation has occurred. The use of this store is necessary to propagate the mutative effect and ensures that its union with the store on which this log is replayed agrees on all common bindings. The effect log of the CALL rule is composed of the effect log of evaluation of the body and an entry for the allocation of the bound variable. Finally, the effect log of the LET rule is composed of the effect logs of evaluation of both the body and binding expression interposed by an entry for the allocation of the bound variable.

In this semantics (and the next), the bindings in $\sigma'$ are redundant: once $\mathsf{extend}_{log}$ applies the the mutative or allocative binding to its argument $\sigma$, $\sigma$ already contains all the bindings of $\sigma'$. Once we introduce GC to the semantics, however, this will no longer be the case.

The intended role of the effect log is captured by the following lemma, which states that one may obtain the resultant store by applying the resultant log to the initial store of evaluation.

**Lemma 1.** *If* $\sigma, \rho, t \vdash e \Downarrow_! (v, \sigma'), \xi$*, then* $\sigma' = \xi(\sigma)$*.*

The proof proceeds straightforwardly by induction on the judgment's derivation.

#### 4.3 Compositional-Store Semantics

The third semantics (seen in Figure 4) shifts the previous semantics from threading the store to treating it compositionally. Under this treatment, evaluation results still consist of a value, store, and effect log, but the store is associated directly to the value—at least conceptually—and not treated as a global effect repository. This alternative role is particularly apparent in the Let rule: the store resulting from evaluation of the bound expression is not extended to be used as the initial store of evaluation of the body. Instead, the effect log resulting from evaluation of the bound expression is applied to the initial store (of the overall let expression). We emphasize this compositional treatment by no longer using numeric subscripts, which suggest "evolution" of the store, and instead using ticks, which suggest distinct (but related) instances.

Let

$$\frac{\sigma, \rho, t \vdash ce \Downarrow_\circ (v', \sigma_{v'}), \xi' \qquad \sigma' = \xi'(\sigma) \qquad (\rho', \sigma'') = \mathsf{extend}(\rho, \sigma', x, t, v', \sigma_{v'}) \qquad \sigma'', \rho', t \vdash e \Downarrow_\circ (v, \sigma_v), \xi}{\sigma, \rho, t \vdash \mathsf{let}\, x = ce\, \mathsf{in}\, e \Downarrow_\circ (v, \sigma_v), \xi \circ \mathsf{extend}_{log}((x, t), v', \sigma_{v'}) \circ \xi'}$$

Call

$$\frac{((\lambda x.e, \rho_0), \sigma_0) = \mathsf{aeval}(\sigma, \rho, ae_0) \qquad (v_1, \sigma_1) = \mathsf{aeval}(\sigma, \rho, ae_1) \qquad t' = (ae_0\; ae_1) :: t \qquad (\rho', \sigma') = \mathsf{extend}(\rho_0, \sigma_0, x, t', v_1, \sigma_1) \qquad \sigma', \rho', t' \vdash e \Downarrow_\circ (v, \sigma_v), \xi}{\sigma, \rho, t \vdash (ae_0\; ae_1) \Downarrow_\circ (v, \sigma_v), \xi \circ \mathsf{extend}_{log}((x, t'), v_1, \sigma_1)}$$

Set!

$$\frac{(v, \sigma_v) = \mathsf{aeval}(\sigma, \rho, ae) \qquad a = (x, \rho(x)) \qquad \sigma' = \sigma_v[a \mapsto v]}{\sigma, \rho, t \vdash \mathsf{set!}\, x\; ae \Downarrow_\circ ((\lambda \mathtt{x}.\mathtt{x}, \bot), \sigma'), \mathsf{extend}_{log}(a, v, \sigma')}$$

Atomic

$$\frac{}{\sigma, \rho, t \vdash ae \Downarrow_\circ \mathsf{aeval}(\sigma, \rho, ae), \lambda\sigma.\sigma}$$

**Fig. 4.** The compositional-store semantics

We use the extend metafunction to bind a value $v$ (with an associated store $\sigma_v$) to a variable $x$ in a given binding context $t$ within a given environment $\rho$ and store $\sigma$, defined

$$\mathsf{extend}(\rho, \sigma, x, t, v, \sigma_v) = (\rho[x \mapsto t], \sigma[(x, t) \mapsto v] \cup \sigma_v)$$

When we extend $\sigma$ with a mapping for $v$, we also copy all of the mappings from $\sigma_v$. This copying will yield a well-formed store since $\sigma[(x,t) \mapsto v]$ and $\sigma_v$ agree on any common bindings.

Although the role of the store has changed, the same lemma holds in this semantics as does in the previous. We repeat it in terms of this semantics.

**Lemma 2.** *If* $\sigma, \rho, t \vdash e \Downarrow_\circ (v, \sigma_v), \xi$, *then* $\xi(\sigma) = \sigma_v$.

Like the previous lemma, its proof can be obtained by induction on the judgment's derivation.

#### 4.4 Compositional-Store Semantics with Garbage Collection

Our final semantics (seen in Figure 5) continues the compositional treatment of the store but GCs stores to remove irrelevant bindings. Under this compositional treatment, the role of the store is to model the fragment of the heap which is reachable from an associated environment: the store of a configuration closes the associated environment and the store of a result closes the environment of the associated value. Accordingly, the root set of reachability used by GC includes the addresses of the closed environment only and, in particular, does not include addresses from the continuation. We define reachability just as we did for GC in Section 3.2, using the $\mathsf{root}_v$ and $\mathsf{root}_\rho$ metafunctions to extract a root set from a value and environment, respectively.

In this semantics, we use a modified atomic evaluation function $\mathsf{aeval}_{gc}$ which garbage-collects the store associated with a value. It is defined

$$\mathsf{aeval}_{gc}(\sigma, \rho, x) = (v, \mathsf{gc}(v, \sigma)) \text{ where } v = \sigma(x, \rho(x))$$
$$\mathsf{aeval}_{gc}(\sigma, \rho, \lambda x.e) = (v, \mathsf{gc}(v, \sigma)) \text{ where } v = (\lambda x.e, \rho|_{\lambda x.e})$$

where $\mathsf{gc}(v, \sigma)$ prunes the unreachable bindings from $\sigma$ with respect to $v$.

This semantics is careful to ensure that each evaluation is performed under a store which contains no values unreachable from the environment via frequent use of the $\mathsf{restrict}$ metafunction. For a given expression $e$, closing environment $\rho$, and closing store $\sigma$, the $\mathsf{restrict}$ metafunction first determines the restriction $\rho|_e$ of $\rho$ to the free variables of $e$ and then the bindings of $\sigma$ reachable from $\rho|_e$; it then garbage-collects the store by pruning unreachable bindings. Formally, $\mathsf{restrict}$ is defined

$$\mathsf{restrict}(e, \rho, \sigma) = (\rho|_e, \mathsf{gc}(\rho|_e, \sigma))$$

where $\mathsf{gc}(\rho, \sigma)$ prunes the unreachable bindings from $\sigma$ with respect to $\rho$.

The Let rule proceeds by first obtaining the restriction of the environment and store with respect to the bound expression $ce$, before evaluating $ce$ under that restriction. The evaluation of $ce$ produces a value $v'$, an associated store $\sigma_{v'}$ which closes only that value, and an effect log $\xi'$. The Let rule then replays the effect log $\xi'$ on the initial store $\sigma$ thereby accumulating any mutation (and allocation on which it depends) which occurred. After replaying the log, it extends the resultant store $\sigma'$ and initial environment $\rho$ with a binding for $v'$ and copies

Let

$$\frac{\begin{array}{c}(\rho_{ce}, \sigma_{ce}) = \mathsf{restrict}(ce, \rho, \sigma) \\ \sigma_{ce}, \rho_{ce}, t \vdash ce \Downarrow_{gc} (v', \sigma_{v'}), \xi' \qquad \sigma' = \xi'(\sigma) \qquad (\rho', \sigma'') = \mathsf{extend}(\rho, \sigma', x, t, v', \sigma_{v'}) \\ (\rho_e, \sigma_e) = \mathsf{restrict}(e, \rho', \sigma'') \qquad \sigma_e, \rho_e, t \vdash e \Downarrow_{gc} (v, \sigma_v), \xi\end{array}}{\sigma, \rho, t \vdash \mathsf{let}\, x = ce \,\mathsf{in}\, e \Downarrow_{gc} (v, \sigma_v), \xi \circ \mathsf{extend}_{log}((x, t), v', \sigma_{v'}) \circ \xi'}$$

Call

$$\frac{\begin{array}{c}((\lambda x.e, \rho_0), \sigma_0) = \mathsf{aeval}_{gc}(\sigma, \rho, ae_0) \qquad (v_1, \sigma_1) = \mathsf{aeval}_{gc}(\sigma, \rho, ae_1) \\ t' = (ae_0\; ae_1) :: t \qquad (\rho', \sigma') = \mathsf{extend}(\rho_0, \sigma_0, x, t', v_1, \sigma_1) \\ (\rho_e, \sigma_e) = \mathsf{restrict}(e, \rho', \sigma') \qquad \sigma_e, \rho_e, t' \vdash e \Downarrow_{gc} (v, \sigma_v), \xi\end{array}}{\sigma, \rho, t \vdash (ae_0\; ae_1) \Downarrow_{gc} (v, \sigma_v), \xi \circ \mathsf{extend}_{log}((x, t'), v_1, \sigma_1)}$$

Set!

$$\frac{\begin{array}{c}(v, \sigma_v) = \mathsf{aeval}_{gc}(\sigma, \rho, ae) \\ a = (x, \rho(x)) \qquad \sigma' = \sigma_v[a \mapsto v]\end{array}}{\sigma, \rho, t \vdash \mathsf{set!}\, x\; ae \Downarrow_{gc} ((\lambda \mathtt{x}.\mathtt{x}, \bot), \bot), \mathsf{extend}_{log}(a, v, \sigma')}$$

Atomic

$$\frac{}{\sigma, \rho, t \vdash ae \Downarrow_{gc} \mathsf{aeval}_{gc}(\sigma, \rho, ae), \lambda\sigma.\sigma}$$

**Fig. 5.** The compositional-store semantics with garbage collection the bindings of its associated store $\sigma_{v'}$. Finally, the extended environment and store are restricted with respect to the body expression $e$ before $e$'s evaluation under them.

The Call rule proceeds by first evaluating the atomic operator and argument expressions. After calculating the new binding context $t'$, the operator value environment and store are extended with the new binding. Before evaluation of the body $e$ commences, the extended environment and store are restricted with respect to it.

The Set! rule atomically evaluates the expression $ae$ producing the assigned value. It returns the identity function which, with an empty environment, is closed by an empty store.

The Atomic rule evaluates an atomic expression with $\mathsf{aeval}_{gc}$.

To connect this semantics to the previous, we show that the addition of GC has no semantic effect by the following lemma.

**Lemma 3.** *If* $\sigma, \rho, t \vdash e \Downarrow_\circ (v, \sigma_v), \xi$ *and* $\sigma' = \mathsf{gc}(\rho|_e, \sigma)$ *then* $\sigma', \rho, t \vdash e \Downarrow_{gc} (v, \sigma'_v), \xi'$ *where* $\sigma'_v = \mathsf{gc}(v, \sigma_v)$.

In prose, this lemma states that two evaluation configurations, identical except that one's store is the other's with unreachable bindings pruned, will yield the same evaluation result: their evaluation will produce the same value and, modulo unreachable bindings, the same closing store.

## 5 Abstract Compositional-Store Semantics with Garbage Collection

We now *abstract* the compositional-store semantics with GC—the final semantics of the preceding section. Abstracting the semantics involves (1) defining a finite counterpart of each component of the evaluation configuration and result and (2) defining a counterpart of each semantic rule in terms of these finite components. With each component of the configuration finite, configurations themselves become finite. Then we show that each abstracted rule *simulates* its counterpart—that it admits the full range of its counterpart's behavior. Doing this for each rule ensures that the abstract semantics includes every behavior included by the exact semantics. Once that's complete, we can directly implement our big-step semantics in an abstract definitional interpreter [1, 18] to obtain our stack-precise CFA with GC.

We begin by abstracting each configuration component.

$$\hat{v} \in \widehat{Val} = \mathcal{P}(Lam \times \widehat{Env}) \qquad \hat{\rho} \in \widehat{Env} = Var \rightharpoonup \widehat{Time}$$
$$\hat{t} \in \widehat{Time} = App^{\leq m} \qquad \hat{a} \in \widehat{Address} = Var \times \widehat{Time}$$
$$\hat{\sigma} \in \widehat{Store} = \widehat{Address} \to \widehat{Val} \qquad \hat{\xi} \in \widehat{Log} = \widehat{Address} \to \widehat{Val}$$

Like its concrete counterpart, an abstract store $\hat{\sigma}$ maps an abstract address to an abstract value. Abstract addresses remain a pair of a variable and binding context, only the context is abstract. An abstract value $\hat{v}$, however, is a *set* of abstract closures rather than a single closure. An abstract closure is a $\lambda$ paired with an abstract environment $\hat{\rho}$ which itself is a finite map from variables to binding contexts. An abstract timestamp $\hat{t}$ is a sequence of at most $m$ application sites, where $m$ is a parameter to the analysis.[8] An abstract log $\hat{\xi}$ is an extensional account of the added and modified store mappings relative to the initial store, and takes the same form of an abstract store itself. We define abstract join, composition, and application operators by

$$\hat{\sigma}_0 \sqcup \hat{\sigma}_1 = \lambda\hat{a}.\hat{\sigma}_0(\hat{a}) \cup \hat{\sigma}_1(\hat{a}) \qquad \hat{\xi}_0 \hat{\circ} \hat{\xi}_1 = \hat{\xi}_0 \sqcup \hat{\xi}_1 \qquad \hat{\xi}(\hat{\sigma}) = \hat{\sigma} \sqcup \hat{\xi}$$

To help show that the abstract semantics simulates the concrete, we make a connection between the state space of the abstract and that of the concrete. We make this connection by means of a polymorphic abstraction function $|\cdot|$[9], defined for all domains except stores by

$$|\rho| = \lambda x.|\rho(x)| \qquad |t| = \lfloor t \rfloor_m \qquad |(\lambda x.e, \rho)| = \{(\lambda x.e, |\rho|)\} \qquad |\xi| = |\xi(\bot)|$$

and for stores by

$$|\sigma| = \lambda\hat{a}. \bigcup_{|a|=\hat{a}} |\sigma(a)|$$

[8] The parameter $m$ is used similarly to the parameter $k$ of $k$-CFA.

[9] The abstraction function is typically accompanied by a complementary *concretization* function to complete a Galois connection. For simplicity here, we leave it incomplete.

Abstracting a store groups entries by their abstracted address in a large set. Abstracting an environment $\rho$ abstracts its range. Abstracting a binding context $t$ takes its at-most-$m$-length prefix. Abstracting a closure produces a singleton of that closure with an abstracted environment. Finally, abstracting a log $\xi$ produces the abstract store that results from apply the log to the empty store $\perp$ and then abstracting.

Figure 6 defines the abstract compositional-store semantics with garbage collection. Structurally, nearly every rule is identical to the exact counterpart that it abstracts; most of the work of abstraction is defining the abstract domains and metafunctions and connecting them to those of the exact semantics. The Call rule differs structurally from its exact counterpart in two notable ways: First, because an abstract value is a set of closures, it applies for each such closure in the operator set. Second, it defines the new binding context $\hat{t}'$ to be the prefix of the application site prepended to the previous abstract time $\hat{t}$ and limited to a length of at most $m$. The abstract $\widehat{\mathsf{aeval}}$ metafunction is defined

$$\widehat{\mathsf{aeval}}(\hat{\sigma}, \hat{\rho}, x) = (\hat{v}, \widehat{\mathsf{gc}}(\hat{v}, \hat{\sigma})) \text{ where } \hat{v} = \hat{\sigma}(\hat{\rho}(x))$$
$$\widehat{\mathsf{aeval}}(\hat{\sigma}, \hat{\rho}, \lambda x.e) = (\hat{v}, \widehat{\mathsf{gc}}(\hat{v}, \hat{\sigma})) \text{ where } \hat{v} = \{(\lambda x.e, \hat{\rho}|_{\lambda x.e})\}$$

We omit the straightforward definitions of the abstract variants of $\widehat{\mathsf{gc}}$, $\widehat{\mathsf{restrict}}$, and $\widehat{\mathsf{extend}}$.

Let

$$\frac{\begin{array}{c}(\hat{\rho}_{ce}, \hat{\sigma}_{ce}) = \widehat{\mathsf{restrict}}(ce, \hat{\rho}, \hat{\sigma}) \\ \hat{\sigma}_{ce}, \hat{\rho}_{ce}, \hat{t} \vdash ce \Downarrow (\hat{v}', \hat{\sigma}_{v'}), \hat{\xi}' \qquad \hat{\sigma}' = \hat{\xi}'(\hat{\sigma}) \qquad (\hat{\rho}', \hat{\sigma}'') = \widehat{\mathsf{extend}}(\hat{\rho}, \hat{\sigma}', x, \hat{t}, \hat{v}', \hat{\sigma}_v') \\ (\hat{\rho}_e, \hat{\sigma}_e) = \widehat{\mathsf{restrict}}(e, \hat{\rho}', \hat{\sigma}'') \qquad \hat{\sigma}_e, \hat{\rho}_e, \hat{t} \vdash e \Downarrow (\hat{v}, \hat{\sigma}_v), \hat{\xi}\end{array}}{\hat{\sigma}, \hat{\rho}, \hat{t} \vdash \mathsf{let}\, x = ce \,\mathsf{in}\, e \Downarrow (\hat{v}, \hat{\sigma}_v), \hat{\xi} \hat{\circ} \hat{\xi}'}$$

Call

$$\frac{\begin{array}{c}(\hat{v}_0, \hat{\sigma}_0) = \widehat{\mathsf{aeval}}(\hat{\sigma}, \hat{\rho}, ae_0) \qquad (\lambda x.e, \hat{\rho}_0) \in \hat{v}_0 \\ (\hat{v}_1, \hat{\sigma}_1) = \widehat{\mathsf{aeval}}(\hat{\sigma}, \hat{\rho}, ae_1) \\ \hat{t}' = \lfloor (ae_0\, ae_1) :: \hat{t} \rfloor_m \qquad (\hat{\rho}', \hat{\sigma}') = \widehat{\mathsf{extend}}(\hat{\rho}_0, \hat{\sigma}_0, x, \hat{t}', \hat{v}_1, \hat{\sigma}_1) \\ (\hat{\rho}_e, \hat{\sigma}_e) = \widehat{\mathsf{restrict}}(e, \hat{\rho}', \hat{\sigma}') \qquad \hat{\sigma}_e, \hat{\rho}_e, \hat{t}' \vdash e \Downarrow (\hat{v}, \hat{\sigma}_v), \hat{\xi}\end{array}}{\hat{\sigma}, \hat{\rho}, \hat{t} \vdash (ae_0\, ae_1) \Downarrow (\hat{v}, \hat{\sigma}_v), \hat{\xi}}$$

Set!

$$\frac{\begin{array}{c}(\hat{v}, \hat{\sigma}_v) = \widehat{\mathsf{aeval}}(\hat{\sigma}, \hat{\rho}, ae) \\ (\_, \hat{\xi}) = \widehat{\mathsf{extend}}(\perp, \perp, x, \hat{\rho}(x), \hat{v}, \hat{\sigma}_v)\end{array}}{\hat{\sigma}, \hat{\rho}, \hat{t} \vdash \mathsf{set!}\, x\, ae \Downarrow (\{(\lambda\mathtt{x}.\mathtt{x}, \perp)\}, \perp), \hat{\xi}}$$

Atomic

$$\frac{}{\hat{\sigma}, \hat{\rho}, \hat{t} \vdash ae \Downarrow \widehat{\mathsf{aeval}}(\hat{\sigma}, \hat{\rho}, ae), \perp}$$

**Fig. 6.** The abstract compositional-store semantics with garbage collection

As a final step before we establish the simulation relationship, we define an ordering on stores (and logs, extending it in the natural way):

$$\hat{\sigma}_0 \sqsubseteq \hat{\sigma}_1 \Leftrightarrow \forall \hat{a} \in \widehat{Address}.\hat{\sigma}_0(\hat{a}) \subseteq \hat{\sigma}_1(\hat{a}) \qquad \hat{v}_0 \sqsubseteq \hat{v}_1 \Leftrightarrow \hat{v}_0 \subseteq \hat{v}_1$$

We formally connect this abstract semantics with the concrete compositional-store semantics given in Section 4.4 by the following abstraction theorem.

**Theorem 1.** *If* $|\sigma| \sqsubseteq \hat{\sigma}$ *and* $|\rho| = \hat{\rho}$ *and* $|t| = \hat{t}$ *and* $\sigma, \rho, t \vdash e \Downarrow_{gc} (v, \sigma_v), \xi$*, then* $\hat{\sigma}, \hat{\rho}, \hat{t} \vdash e \hat{\Downarrow} (\hat{v}, \hat{\sigma}_v), \hat{\xi}$ *where* $|v| \sqsubseteq \hat{v}$ *and* $|\sigma_v| \sqsubseteq \hat{\sigma}_v$ *and* $|\xi| \sqsubseteq \hat{\xi}$*.*

This theorem states that if the configuration components are related by abstraction, then, for any given derivation in the exact semantics, there is an derivation in the abstract semantics which yields an abstraction of its results. It can be proved by induction on the derivation.

### 6 Discussion

Now we examine the ramifications of a compositional treatment of analysis components. We do so in turn, first considering the ramifications of treating the store compositionally and then of treating the time compositionally.

#### 6.1 The Effects of Treating the Store Compositionally

We saw in Section 4.3 that a semantics could treat stores compositionally without employing GC. In this case, the caller's store and callee's final store agreed on common entries and combining them produced the same store as the threaded-store semantics. However, the compositional machinery liberates evaluation from the stack. With evaluation so-liberated, GC need not preserve any heap data reachable solely from the stack. This relaxation

1. simplifies GC and increases its effectiveness;
2. leads to general yet precise summaries; and
3. restores context irrelevance under GC.

We discuss each of these aspects in more detail.

**Simplified and More-Effective Garbage Collection** Classical abstract GC and its succeeding pushdown GC each preserve heap data reachable from both the local environment and the stack. Once one has determined the root set of reachable addresses from these two components, it determines the transitive closure of reachability. When GC is performed with respect to only the local environment, both the initial root set and its transitive closure are smaller and it requires less work to calculate them. If the CFA employs incomplete garbage collection [8], the garbage collector is also freed from calculating the root set of stack addresses as a fixed point. A smaller transitive closure of reachable addresses is not only less costly to calculate but also leads to more collected garbage.

**General Yet Precise Summaries** A stack-precise CFA without GC will falsely distinguish abstract evaluations of the same call which are identical modulo GC-able heap data. In such cases, the addition of pushdown GC will allow the CFA to identify them. However, even with pushdown GC, a stack-precise CFA will falsely distinguish abstract evaluations of the same call which are identical modulo continuation-reachable heap data. On the other hand, compositional GC soundly disregards such data and thereby identifies such evaluations.

Compositional GC is able to achieve this feat because its calculates the fragments of the heap reachable from the local environment alone. Since this environment is restricted to the free variables of the expression it closes, the resultant heap fragment includes a tight overapproximation of the actually-relevant heap data. One effect is that evaluation summaries—the association of an evaluation configuration with its results—are general yet precise. They are general since, with a minimum of irrelevant heap data, more contexts are consistent with them. They are precise since, with a minimum of irrelevant heap data, they are less likely to allocate an entry at an existing address. In fact, the precision of compositional GC dominates that of pushdown GC.

**Restored Context Irrelevance** A semantics determines which parts of a given configuration are *relevant* to its evaluation [8]. When the continuation is irrelevant to evaluation, the semantics exhibits the property of *context irrelevance*. Context irrelevance is an intuitive property: unless our semantics has control effects or some other explicit dependence, we would be surprised if a configuration's continuation was relevant to its evaluation. Even a concrete semantics with GC exhibits context irrelevance since data reachable from the stack alone will not effect the result of evaluation. In an abstract semantics with GC, however, where new allocations can occur at old addresses, the presence of data reachable from the stack alone can affect evaluation. The set of data preserved by GC, which determines how evaluation is affected, is itself determined by the continuation. Thus, an abstract semantics in which GC is defined *with respect to the stack* violates context irrelevance.

Put this way, it is clear why compositional GC restores context irrelevance to the semantics: it removes the dependence on the stack from GC itself and allows all data reachable from the stack alone to be collected. This restoration makes evaluation easier to reason about and increases the effectiveness of memoization.

#### 6.2 The Effect of Treating the Time Compositionally

The $k$-CFA context abstraction consists of a sequence of $k$ call sites—for each point in execution, the last $k$ call sites encountered. In Section 3.5, we discussed how the last-$k$-call-sites abstraction arose as a consequence of the semantics threading the abstract time (i.e. the context) through execution.

In contrast, the big-step, concrete semantics of Section 4 and the big-step, abstract semantics of Section 5 didn't thread the abstract time through execution but treated it compositionally, installing a new time at a call but restoring the previous time at the corresponding return. This treatment of time induces a different notion of context than $k$-CFA; instead of yielding the last-$k$ call sites, it yields the top-$m$ stack frames.

This top-$m$-stack-frames context abstraction is not novel and originates with $m$-CFA [11], a family of polynomial-time CFAs. However, to our knowledge, its appearance here is its first in a stack-precise setting: many stack-precise CFAs encode context using other means than a time component (or don't use context in the first place) [16, 3, 1]; still others achieve the last-$k$-call-sites abstraction, incidentally or intentionally [4, 18].

Using the top-$m$ stack frames to qualify heap allocation has certain advantages to using the last-$k$ call sites; in particular, its power to distinguish bindings is not diluted by static call sequences. To see how $k$-CFA's and $m$-CFA's context abstractions compare, let's consider a few examples.

First, consider a $[k = 2]$CFA of the program

```
(define (f x) x)
(define (g y) (f y))
(g 42)
(g 35)
```

the abstract resource 42 is allocated in the heap twice—first when the call to `g` is made and second when the call to `f` is made. At the point of the second allocation, the two most-recently-encountered call sites in evaluation are `(f y)` and `(g 42)`; hence, these call sites are used to qualify the binding of 42 to `x` in the heap. The treatment of the abstract resource 35 is similar except its second allocation is qualified by `(f y)` and `(g 35)`. For this program, $[k = 2]$CFA is able to keep the two allocations distinct.

Next, consider a $[k = 2]$CFA of the similar program

```
(define (f x) x)
(define (g y)
  (displayln y)
  (f y))
(g 42)
(g 35)
```

which includes the call `(displayln y)` in the body of `g`. As in the previous program, the analysis of this program allocates the abstract resources 42 and 35 twice each. However, in this program, the second of each of their allocations is qualified by `(f y)` and `(displayln y)`. In fact, every call to `f` made via `g` will occur in that same context. In a sense, the static sequence of `(displayln y)` and `(f y)` eats up the context budget ensuring that the analysis conflates all bindings made at the call `(f y)`. (Incrementing $k$ would remove the conflation in this example, but it makes the analysis more expensive and such a strategy can always be confounded by a longer "static" trace of calls.)

To constrast, consider an $[m = 2]$CFA of the same program. Because the context consists of the top two stack frames, the allocation of 42 is qualified by

`(f y)` and `(g 42)` and the allocation of 35 is qualified by `(f y)` and `(g 35)`. Because the second stack frame of each allocation is distinct, $[m = 2]$CFA is able to keep the bindings distinct in the analysis.

The top-$m$-stack-frames context abstraction is itself susceptible to deep nests of calls which serve only to pass parameters: if the nesting depth exceeds $m$, then the analysis will conflate the bindings made by the innermost calls. And, as with $k$-CFA, an increased $m$ can always be confounded by a deeper nesting. In spite of that, the $m$-CFA context abstraction has been shown to work well relative to $k$-CFA in practice in a stack-imprecise setting where variables are aggressively re-bound [11]. Future work is needed to verify that its advantages carry over to a stack-precise setting.

### 7 Related Work

Broadly, this work is an instance of abstract interpretation and, more specifically, of control-flow analysis (CFA) [9, 14]. It inherits from the *Abstracting Abstract Machines* methodology [15] of systematically deriving CFAs from purely operational specifications. More specifically, this work is an instance of stack-precise CFA which is preceded by many variations [16, 3, 8, 6, 12, 1, 18].

Might and Shivers [10] first introduced GC to CFA. Reconciling GC with stack-precise CFAs has been the focus of significant effort. Earl *et al.* [4] introduced the first technique to do so which approximated the the set of frames that could be on any possible stack at any given control point. Johnson and Van Horn [8] cast this technique into a more operational framework and considered a more-precise variant in which a control point splits for each possible stack with its heap being collected with respect to that stack alone. Johnson *et al.* [7] unified these previous two works in one formal framework. Darais *et al.* [1] show that the *Abstracting Definitional Interpreters* approach easily accommodates abstract GC by introducing a machine component which contains the addresses embedded in stack frames; this realization of GC amounts essentially to the fully-precise technique. Our work sidesteps the need for all of this previous effort by decomposing the heap into continuation-independent fragments.

A significant concept in the work of Johnson and Van Horn [8] is *context irrelevance*, the property that the evaluation of a configuration is independent of its continuation, and they note that the approximate abstract GC technique introduced by Earl *et al.* [4] violates context irrelevance. Once again, the independence of GC from the stack under our technique sidesteps these issues; evaluation under our technique exhibits context irrelevance effortlessly.

As part of the resolution of an apparent paradox regarding the complexities of object-oriented $k$-CFA and functional $k$-CFA, Might *et al.* [11] develop $m$-CFA, a stack-imprecise, polynomial-time family of CFA that employs the top-$m$ stack frames as a context abstraction as opposed to the last-$k$ call sites of $k$-CFA. They show that this abstraction is more resilient against approximation in the face of the aggressive rebinding that $m$-CFA effects. Our treatment of the abstract time component induces this same top-$m$-stack-frames context abstraction but in a stack-precise setting, the first such appearance in the literature, to our knowledge.

Although not inspired by it, our work surprisingly shares much of the perspective and approach of the work of Dillig *et al.* [2] to verify C and C++ programs. In particular, both works employ a compositional approach to analysis by producing evaluation summaries and decompose the heap to support their approach. In addition, both works have some notion of propagation of summary effects: theirs is a *summary transfer function*; ours is an effect log. In contrast, our work does not produce summaries in a bottom-up fashion and is targeted toward explicitly higher-order languages with effects. Interesting future work could explore whether any precision-enhancing techniques of Dillig *et al.* [2] could be ported and applied, whether the bottom-up production of summaries is viable, or whether their general approach can be used for verification in our setting.

### 8 Conclusion and Future Work

In this paper, we showed that treating the heap compositionally in a stack-precise CFA removes its dependence on the stack, at once simplifying GC and increasing its effectiveness. As a result, the analysis produces more compact and precise evaluation summaries that are more amenable to reuse. We also showed that treating the time component compositionally induces the top-$m$-stack-frames context abstraction of $m$-CFA. Unlike $k$-CFA's last-$k$-call-sites context abstraction, $m$-CFA's need not devote any precision to static call sequences.

Interestingly, the notion of context shared by $k$-CFA and $m$-CFA—calling context, roughly—seems to be at odds with summary reuse. In a stack-precise 1CFA (which exhibits the same context abstraction whether it is $[k = 1]$CFA or $[m = 1]$CFA), the syntactic call site of the caller is encoded in the summary of the callee, preventing the summary's reuse at any other call site. If this tension is fundamental, it might benefit to look to alternative notions of context—extant and novel.

The complement to abstract GC is abstract counting [10] which keeps track of the number of concrete resources that correspond to an abstract resource and enables certain abstract transitions, such as a strong store update. If an abstact counting can be applied to heap fragments such that the overlap among fragments is accounted for correctly, it might be possible to detect opportunities to perform strong updates to heap bindings which would further increase the precision of our technique.

Finally, Darais *et al.* [1] consider a particular value abstraction in which primitive operations propagate imprecision but do not introduce it. Their abstraction suggests a generalization in which each "basic block" is analyzed at full precision and imprecision occurs only at the join points of control flow. CFA2's stack environments capture an aspect of this generalization and it appears our technique does as well. However, a focused investigation would reveal whether such a generalization can be more-fully realized.

## References

[1] Darais, D., Labich, N., Nguyen, P.C., Van Horn, D.: Abstracting definitional interpreters (functional pearl). Proceedings of the ACM on Programming Languages **1**(ICFP), 12:1–12:25 (Aug 2017). 10.1145/3110256

[2] Dillig, I., Dillig, T., Aiken, A., Sagiv, M.: Precise and compact modular procedure summaries for heap manipulating programs. In: Proceedings of the 32Nd ACM SIGPLAN Conference on Programming Language Design and Implementation. pp. 567–577. PLDI ’11, ACM, New York, NY, USA (2011). 10.1145/1993498.1993565

[3] Earl, C., Might, M., Van Horn, D.: Pushdown control-flow analysis of higher order programs. Workshop on Scheme and Functional Programming (2010)

[4] Earl, C., Sergey, I., Might, M., Van Horn, D.: Introspective pushdown analysis of higher-order programs. In: Proceedings of the 17th ACM SIGPLAN International Conference on Functional Programming. pp. 177–188. ICFP ’12, ACM, New York, NY, USA (Sep 2012). 10.1145/2364527.2364576

[5] Flanagan, C., Sabry, A., Duba, B.F., Felleisen, M.: The essence of compiling with continuations. In: Proceedings of the ACM SIGPLAN 1993 Conference on Programming Language Design and Implementation. pp. 237–247. PLDI ’93, ACM, New York, NY, USA (1993). 10.1145/155090.155113

[6] Gilray, T., Lyde, S., Adams, M.D., Might, M., Van Horn, D.: Pushdown control-flow analysis for free. In: Proceedings of the 43rd Annual ACMSIGPLAN-SIGACT Symposium on Principles of Programming Languages. pp.691–704. POPL ’16, ACM, New York, NY, USA (Jan 2016). 10.1145/2837614.2837631

[7] Johnson, J.I., Sergey, I., Earl, C., Might, M., Van Horn, D.: Pushdown flow analysis with abstract garbage collection. Journal of Functional Programming **24**, 218–283 (May 2014). 10.1017/s0956796814000100

[8] Johnson, J.I., Van Horn, D.: Abstracting abstract control. In: Proceedings of the 10th ACM Symposium on Dynamic Languages. pp. 11–22. DLS ’14, ACM, New York, NY, USA (Oct 2014). 10.1145/2661088.2661098

[9] Jones, N.D.: Flow analysis of lambda expressions. In: International Colloquium on Automata, Languages, and Programming. pp. 114–128. Springer (1981)Jones, N.D.: Flow analysis of lambda expressions. In: International Colloquium on Automata, Languages, and Programming. pp. 114–128. Springer (1981)

[10] Might, M., Shivers, O.: Improving flow analyses via $$\varGamma $$CFA: abstract garbage collection and counting. In: Proceedings of the Eleventh ACM SIGPLAN International Conference on Functional Programming. pp. 13–25. ICFP ’06, ACM, New York, NY, USA (Sep 2006). 10.1145/1159803.1159807

[11] Might, M., Smaragdakis, Y., Van Horn, D.: Resolving and exploiting the *k*-CFA paradox: illuminating functional vs. object-oriented program analysis. In: Proceedings of the 31st ACM SIGPLAN Conference on Programming Language Design and Implementation. pp. 305–315. PLDI ’10, ACM, New York, NY, USA (Jun 2010). 10.1145/1806596.1806631

[12] Peng, F.: $$h$$-CFA: A simplified approach for pushdown control flow analysis. Master’s thesis, The University of Wisconsin-Milwaukee (2016)

[13] Reynolds, J.C.: Definitional interpreters for Higher-Order programming languages. Higher-Order and Symbolic Computation **11**(4), 363–397(1998)

[14] Shivers, O.: Control-Flow Analysis of Higher-Order Languages. Ph.D. thesis,Carnegie Mellon University, Pittsburgh, PA, USA (1991)

[15] Van Horn, D., Might, M.: Abstracting abstract machines. In: Proceedings of the 15th ACM SIGPLAN International Conference on Functional Programming. pp. 51–62. ICFP ’10, ACM, New York, NY, USA (Sep 2010). 10.1145/1863543.1863553

[16] Vardoulakis, D., Shivers, O.: CFA2: A context-free approach to control-flow analysis. In: Gordon, A.D. (ed.) Programming Languages and Systems. pp. 570–589. Springer Berlin Heidelberg, Berlin, Heidelberg (2010)

[17] Vardoulakis, D., Shivers, O.: CFA2: a context-free approach to control-flow analysis. Logical Methods in Computer Science **7**(2) (2011). 10.2168/LMCS-7(2:3)2011

[18] Wei, G., Decker, J., Rompf, T.: Refunctionalization of abstract abstract machines: bridging the gap between abstract abstract machines and abstract definitional interpreters (functional pearl). Proceedings of the ACM on Programming Languages **2**(ICFP), 105:1–105:28 (Jul 2018). 10.1145/3236800

